# Genomic analyses of unique carbohydrate and phytohormone metabolism in the macroalga *Gracilariopsis lemaneiformis* (Rhodophyta)

**DOI:** 10.1186/s12870-018-1309-2

**Published:** 2018-05-25

**Authors:** Xue Sun, Jun Wu, Guangce Wang, Yani Kang, Hong Sain Ooi, Tingting Shen, Fangjun Wang, Rui Yang, Nianjun Xu, Xiaodong Zhao

**Affiliations:** 1 Key Laboratory of Marine Biotechnology of Zhejiang Province, School of Marine Sciences, Ningbo University, Ningbo, 315211 People’s Republic of China; 2 School of Biomedical Engineering, Shanghai Center for Systems Biomedicine, Shanghai Jiao Tong University, Shanghai, 200240 People’s Republic of China; 3 Institute of Oceanology, Chinese Academy of Sciences, Qingdao, 266071 People’s Republic of China; 4 Department of Biomedicine, Aarhus University, 8000 Aarhus C, Denmark

**Keywords:** *Gracilariopsis lemaneiformis*, Genomic analysis, Carbohydrate metabolism, Phytohormone signaling

## Abstract

**Background:**

Red algae are economically valuable for food and in industry*.* However, their genomic information is limited, and the genomic data of only a few species of red algae have been sequenced and deposited recently. In this study, we annotated a draft genome of the macroalga *Gracilariopsis lemaneiformis* (Gracilariales, Rhodophyta).

**Results:**

The entire 88.98 Mb genome of *Gp. lemaneiformis* 981 was generated from 13,825 scaffolds (≥500 bp) with an N50 length of 30,590 bp, accounting for approximately 91% of this algal genome. A total of 38.73 Mb of scaffold sequences were repetitive, and 9281 protein-coding genes were predicted. A phylogenomic analysis of 20 genomes revealed the relationship among the Chromalveolata, Rhodophyta, Chlorophyta and higher plants. Homology analysis indicated phylogenetic proximity between *Gp. lemaneiformis* and *Chondrus crispus*. The number of enzymes related to the metabolism of carbohydrates, including agar, glycoside hydrolases, glycosyltransferases, was abundant. In addition, signaling pathways associated with phytohormones such as auxin, salicylic acid and jasmonates are reported for the first time for this alga.

**Conclusion:**

We sequenced and analyzed a draft genome of the red alga *Gp. lemaneiformis*, and revealed its carbohydrate metabolism and phytohormone signaling characteristics. This work will be helpful in research on the functional and comparative genomics of the order Gracilariales and will enrich the genomic information on marine algae.

**Electronic supplementary material:**

The online version of this article (10.1186/s12870-018-1309-2) contains supplementary material, which is available to authorized users.

## Background

Red algae (or Rhodophyta) compose an ancient and unique clade of photosynthetic eukaryotes that includes more than 6500 species [1]. Red algae have important economic value as raw materials in the food, medicine and phycocolloid industries. Additionally, red algae have received increasing attention from the scientific community due to their genetic contributions to other eukaryotic organisms through secondary endosymbiosis, enabling the tracking of the evolutionary history of many eukaryotic lineages [2]. However, relatively little is known about the genomic and genetic backgrounds of red algae.

The red alga *Gracilariopsis lemaneiformis* (*Gp. lemaneiformis*), formerly known as *Gracilaria lemaneiformis* (*G. lemaneiformis*) [3], was previously thought to be distributed in China, Japan, Peru, and America. However, Gurgel et al. [4] reported *Gp. lemaneiformis* was not distributed worldwide and was restricted to the vicinity of Peru. In China, *Gp. lemaneiformis* has become the third largest cultivated seaweed after *Saccharina* and *Pyropia*, and has an annual dry weight of 246 million kg. Wild populations of *Gp. lemaneiformis* are mainly distributed along the northern coast of China, but the high-temperature-tolerant cultivar 981 can be cultivated from the northern to the southern coasts [5]. The cultivated *Gp. lemaneiformis* are used as a delicious food source for humans, as abalone feed and as a resource for producing agar. In addition, *Gp. lemaneiformis* is effective at removing nitrogen and phosphorus from seawater and inhibiting red tide causing microalgae [6, 7].

The red alga *Gp. lemaneiformis* contains abundant and diverse carbohydrates such as polysaccharide and agar. The polysaccharides of the algae have antitumor, hypoglycemic and immunomodulatory activities [8–10]. Agar, one of the main carbohydrates in *Gp. lemaneiformis*, has diverse applications in the food, pharmaceutical, cosmetic, medical and biotechnology industries. Approximately 9600 tons of agar were produced in 2009 (US$173 million), of which 80% was obtained from *Gracilaria* or *Gracilariopsis* [11]. Although many species of the Gracilariales are good candidates for commercial agar extraction, *Gp. lemaneiformis* is superior because of its high agar content [12].

Phytohormones, or plant hormones, play a key role in plant growth and development or act as regulators of the defense response against adverse environmental conditions. Phytohormones have similar functions in algae and higher plants [13]. Many phytohormones, including indole-3-acetic acid (IAA), abscisic acid (ABA), jasmonic acid (JA) and salicylic acid (SA), have been detected in *Gp. lemaneiformis* [14]. However, the phytohormone signaling pathways in algae may be different from those in higher plants.

Up to now, limited information is available regarding the genetic architecture, transcribed genes and metabolic pathways in *Gp. lemaneiformis*. Although the mitochondrial and chloroplast structures of *Gp. lemaneiformis* have been investigated [15, 16], and although some genomic information is available [17], the limited knowledge regarding this species has impeded genetic studies and cultivation practices. In this study, we report a draft genome of *Gp. lemaneiformis* using the high-throughput sequencing technology. Then, we discuss the metabolic pathways of agar and other carbohydrates, as well as phytohormone signaling pathways. Our work will offer an invaluable resource for *Gp. lemaneiformis* and studies on other red algae.

## Results

### Genome sequencing and assembly

**Fig. 1:**
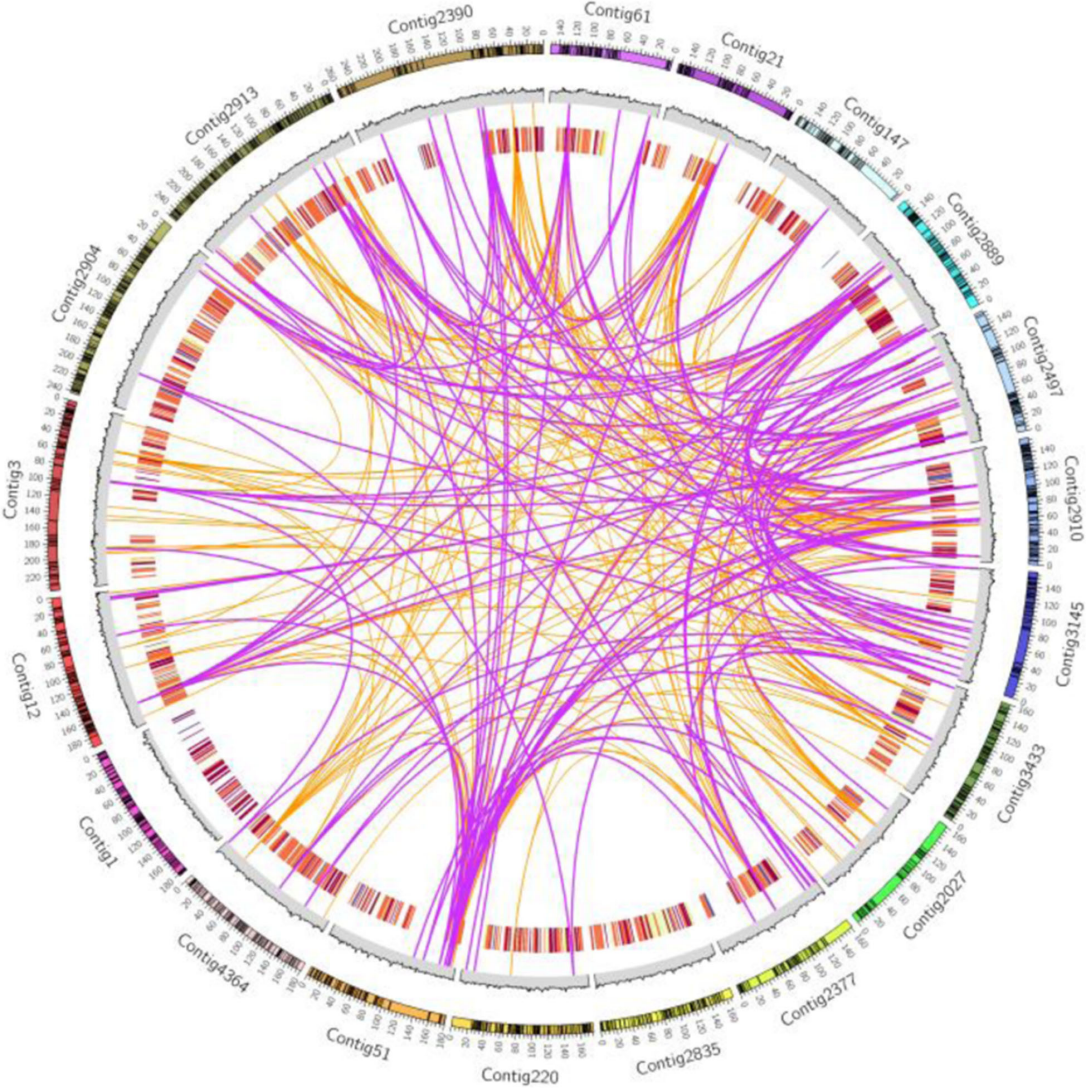
Circular diagram depicting the genomic features of the top 20 longest contigs. The outer ring represents the top 20 longest contigs, and the black blocks represent the predicted genes. The middle ring shows the GC content of the corresponding contigs in 1 kb bins. The inner rings show expression of the predicted genes. The blue color indicates low expression, and the red color indicates high expression. The links in the plot represent similarity between the contigs

In this paper, we assembled a draft genome of 88.98 Mb from tetrasporophytic thalli of *Gp. lemaneiformis* 981. Approximately 60.7 million paired-end sequencing reads (2×100 bp) were generated using an Illumina HiSeq sequencer. Simultaneously, 623.6 million reads with an average length of 4305 bp were generated using a Pacific Biosciences sequencer. These raw sequencing reads, which represent approximately 189-fold coverage based on the estimated *Gp. lemaneiformis* genome size [18], were combined for the genome assembly. Using 62,208 contigs with an N50 of 28,502 bp, 92.58 Mb of sequences were assembled. A total of 48,035 scaffolds yielded a 94.60 Mb genome with an N50 length of 30,590 bp. Ultimately, an 88.98 Mb genome derived from 13,825 scaffolds with lengths equal to or greater than 500 bp was used for further analysis (Table 1). The GC content of the *Gp. lemaneiformis* genome was 48.13%. The top 20 assembled contigs with the longest and most genes and the corresponding RNA abundance are shown in Fig. 1.

**Table 1:** Summary of the *Gp. lemaneiformis* genome assembly

	Contigs	Scaffolds
N50 (bp)	28,502	30,590
Longest (bp)	525,375	525,375
Total number	62,208	48,035
Total size (Mb)	92.58	94.60
Total number (≥500 bp)	–	13,825
Total size (Mb) (≥500 bp)	–	88.98

To determine whether the assembled *Gp. lemaneiformis* genome matched the published data, we mapped the scaffolds to the published mitochondrial genome of *Gp. lemaneiformis* [15]. We found that two scaffolds (i.e., scaffold2118 with a length of 13,296 bp, and scaffold2119 with a length of 13,237 bp) fully aligned to the mitochondrial genome, suggesting that the assembly reported here was reliable.

### Genome annotation and gene prediction

**Fig. 2:**
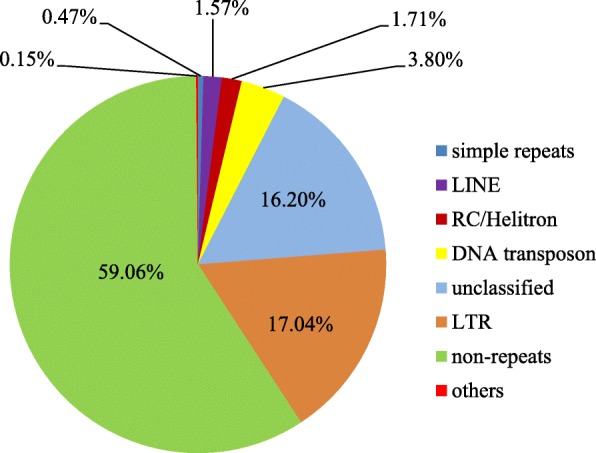
Repetitive element distribution in the *Gp. lemaneiformis* genome. LINEs, long interspersed repeated DNA elements. LTR, long terminal repeat; RC/Helitron, rolling-circle transposon

Using homology-based and ab initio prediction approaches, we generated a repetitive element dataset, which was used to annotate the *Gp. lemaneiformis* genome. This investigation led to the identification of 38.73 Mb of repetitive sequences, accounting for 40.94% of the total assembled algal genome. Except for unclassified repeats, approximately 60.45% of the repeats were classified into known families (Fig. 2). Class I, long terminal repeat (LTR) retrotransposons, were found to be the most abundant repeat elements (17.04% of the entire genome), representing 41.60% of the total known repeat sequences, including the Gypsy and Copia families. Class II, DNA transposons, were the second most abundant repeat family, accounting for 3.80% of the genome. In addition to cut-and-paste class II transposable elements (TEs), we identified 1.71% of rolling-circle transposons (RC/Helitron) in this algal genome. Helitrons are transposons that function via a rolling-circle replication mechanism and have been reported in > 2% of *Arabidopsis thaliana* genomes [19].

To accurately annotate the protein-coding genes in the genome of *Gp. lemaneiformis*, we used a combined strategy that integrated gene predictions, protein-homolog-based approaches and transcriptome-evidence-based approaches. In total, we predicted 9281 genes with an average length of 1398 bp in the algal genome. Approximately 62.40% of the predicted coding loci were supported by the RNA-seq data. The results demonstrate the high accuracy of gene prediction in the *Gp. lemaneiformis* genome.

### Comparative genomics

**Fig. 3:**
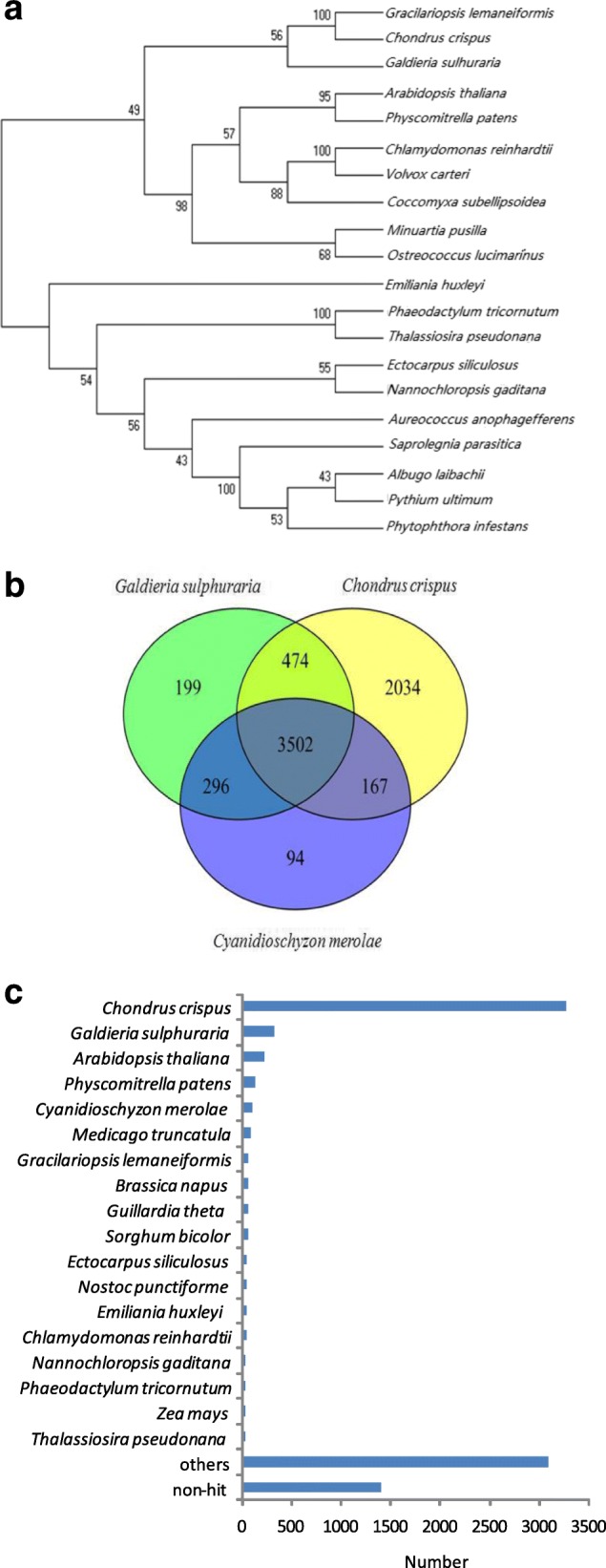
Comparative genomic analysis of *Gp. lemaneiformis*. **a** The phylogenetic tree was generated using the maximum likelihood method based on single-copy genes shared between algal and plant genomes. **b** The Venn diagram represents the *Gp. lemaneiformis* genes shared in the *C. crispus*, *C. merolae* and *G. sulphuraria* genomes. **c** Gene models of *Gp. lemaneiformis* are compared with the characterized genomes in the non-redundant protein database using BLASTp. The number of organisms with the top BLASTp hits against *Gp. lemaneiformis* is indicated

A total of 20 genomes from the Chromalveolata, Rhodophyta, Chlorophyta and higher plants were selected for phylogenomic analysis, and the single-copy genes that were shared by these species were used to generate a phylogenetic tree. As shown in Fig. 3a, the 20 species were divided into the following two main groups in the tree: Archaeplastida and Chromalveolata. The Archaeplastida contained red algae, green algae and higher plants. According to the phylogenetic tree, *Gp. lemaneiformis* is phylogenetically close to *C. crispus*, which is a multicellular red alga with a fully sequenced genome.

To investigate the features of the *Gp. lemaneiformis* genome, we examined the genes that were homologous to those of three other red algae. Of the four species, the marine macroalgae *Gp. lemaneiformis* and *C. crispus* belonged to the Florideophyceae; however, *Cyanidioschyzon merolae* and *Galdieria sulphuraria* are closely related unicellular thermo-acidophilic red algae (Bangiophyceae and Cyanidiaceae). In total, 6177, 4059, and 4471 common genes were identified after comparing *Gp. lemaneiformis* with *C. crispus*, *C. merolae*, and *G. sulphuraria*, respectively*.* Overall, 3502 genes were common across these 4 species (Fig. 3b). The results of this analysis confirmed the phylogenetic proximity between *Gp. lemaneiformis* and *C. crispus* and revealed the broader evolutionarily relationship between macroalgae and unicellular red algae.

In addition, we compared the predicted gene models in the non-redundant protein database of algae and plants using the best-hit method. This analysis yielded top hits from various organisms, and *C. crispus*, *G. sulphuraria* and *A. thaliana* were the most frequent species (Fig. 3c).

### Gene ontology (GO) and Kyoto encyclopedia of genes and genomes (KEGG) analyses

**Fig. 4:**
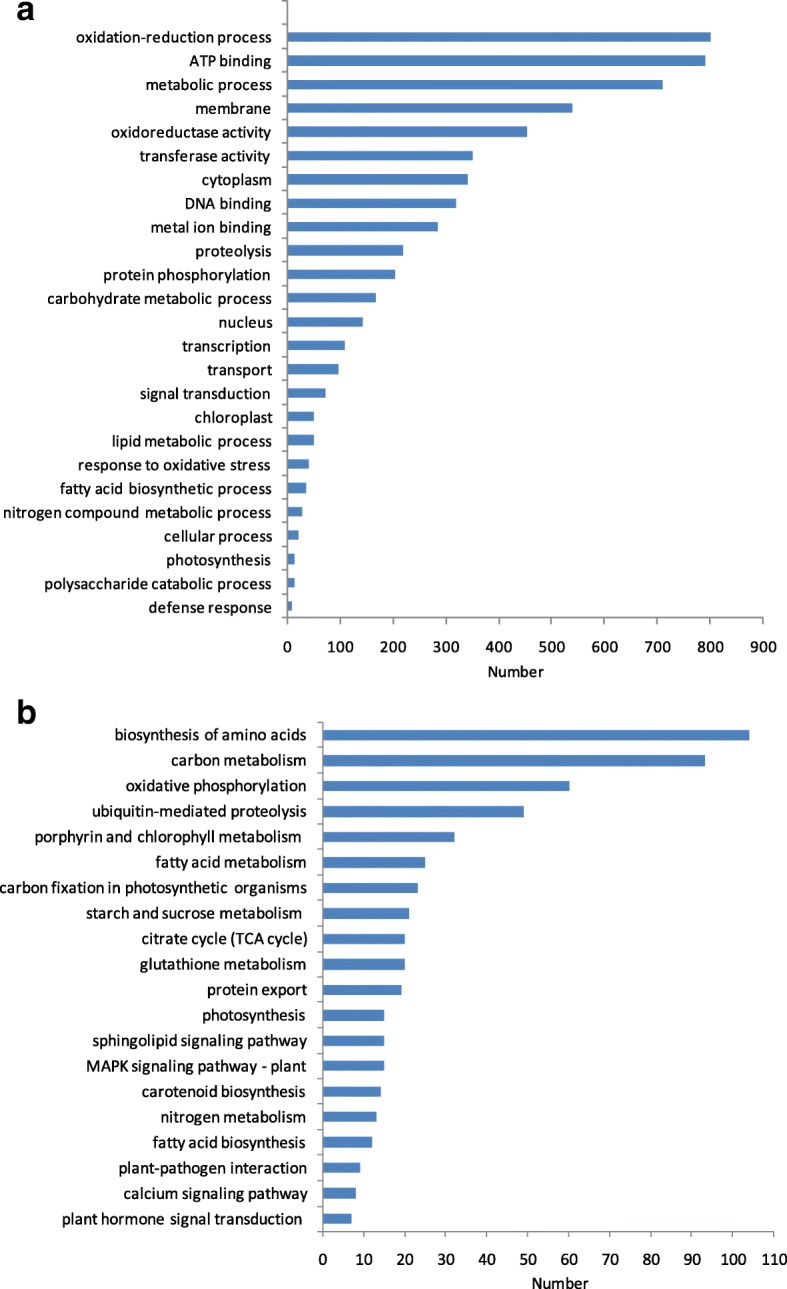
GO and KEGG analyses of the *Gp. lemaneiformis* genome. **a** GO terms derived from the *Gp. lemaneiformis* gene models. **b** KEGG pathway lists for *Gp. lemaneiformis*

To investigate the genes and pathways that are specifically involved in this alga, we performed GO and KEEG analyses using the gene models predicted in this study. Based on the three categories biological process (BP), cellular component (CC), and molecular function (MF), we found that the most frequent GO terms included “oxidation-reduction process” (BP), “ATP binding” (MF), “metabolic process” (BP), “membrane” (CC), “oxidoreductase activity” (MF), “transferase activity” (MF) and “cytoplasm” (CC) (Fig. 4a). In the KEGG analysis, the pathways “biosynthesis of amino acids”, “carbon metabolism”, and “oxidative phosphorylation” were enriched, containing 104, 93 and 60 genes in the *Gp. lemaneiformis* genome, respectively (Fig. 4b).

### Carbohydrate metabolism analysis

#### Agar biosynthesis-related enzymes

**Fig. 5:**
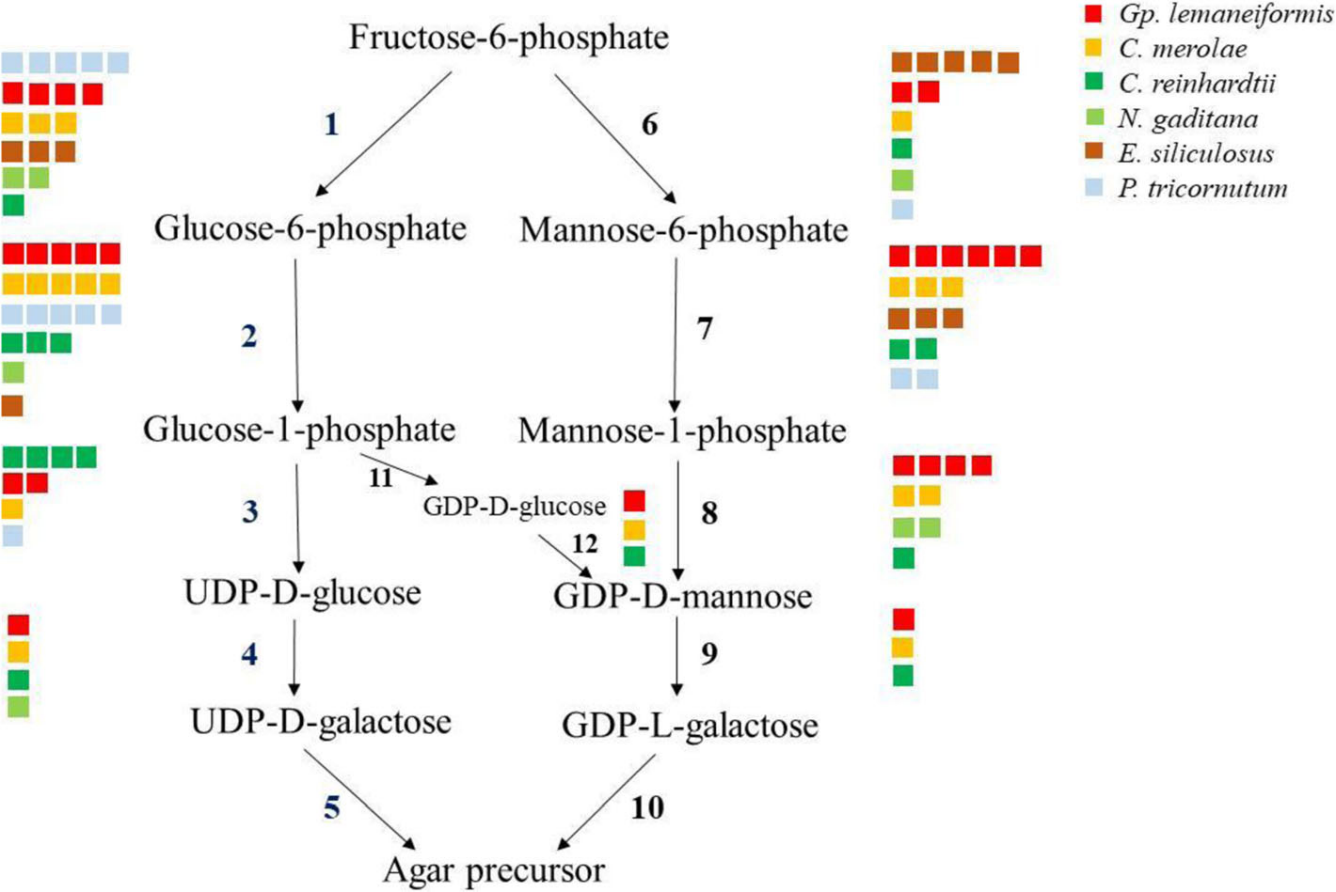
Analysis of genes potentially involved in agar biosynthesis. Gene homologs in the agar biosynthetic pathways in *Gp. lemaneiformis* compared with those in *C. merolae*, *N. gaditana*, *E. siliculosus*, *C. reinhardtii* and *P. tricornutum*. The colored squares denote the number of homologous genes in each species. The step numbers and their representative enzymes are **1.** phosphoglucose isomerase; **2.** phosphoglucomutase; **3.** UTP-glucose-1-phosphate uridylyltransferase; **4.** galactose-1-phosphate uridylyltransferase; **5.** UDP galactosyltransferase; **6.** phosphomannose isomerase; **7.** phosphomannomutase; **8.** GTP-mannose-1-phosphate guanylyltransferase; **9.** GDP-mannose-3,5-epimerase; **10.** GDP galactosyltransferase; **11.** UDP-glucose pyrophosphorylase; **12.** GDP-mannose-3,5-epimerase (the same as **9**)

Based on the agar biosynthetic pathway in red algae [20, 21], we compared the homologous genes involved in agar precursor biosynthesis in *Gp. lemaneiformis* with those in other algae, including the red alga *C. merolae*, the green alga *Chlamydomonas reinhardtii*, the stramenopile alga *Nannochloropsis gaditana*, the brown alga *Ectocarpus siliculosus*, and the diatom *Phaeodactylum tricornutum* (Fig. 5 and Additional file 1: Table S1). Among these six species of algae, the enzymes phosphomannomutase (**7**) and GTP-mannose-1-phosphate guanylyltransferase (**8**) were the most abundant in *Gp. lemaneiformis*, and phosphoglucomutase (**2)** was also highly enriched, to the same degree as in *C. merolae* or *P. tricornutum*. The numbers of three enzymes (**4**, **9** and **12**) in *Gp. lemaneiformis* were equal to those in several species. However, the degree of enrichment of phosphoglucose isomerase (alternatively known as glucose-6-phosphate isomerase, **1**), UTP-glucose-1-phosphate uridylyltransferase (also known as UDP-glucose pyrophosphorylase, **3**) and phosphomannose isomerase (alternatively mannose-6-phosphate isomerase, **6**) in *Gp. lemaneiformis* was second to their enrichment in *P. tricornutum* (**1**), *C. reinhardtii* (**3**) and *E. siliculosus* (**6**), respectively. Although enzymes **5**, **10** and **11** were not detected, *Gp. lemaneiformis* had an expanded repertoire of agar biosynthesis-related enzymes.

After the agar precursor is synthesized, a set of genes, including methyltransferases (MTs), sulfotransferases (STs), sulfurylases, pyruvyl transferases (PTs), sulfatases and sulfohydrolases (SHs), are needed to convert the agar precursor to agar [21].

Sulfotransferases are responsible for catalyzing the transfer of a sulfur group from a donor to an acceptor alcohol or amine molecule [22]. In *Chondrus*, 12 ST-encoding genes have been identified [23]; however, we found eight STs in *Gp. lemaneiformis* (Additional file 2: Table S2). Of the eight ST-encoding genes, three contigs (Contig145.10, 219.13 and 4885.26) encode carbohydrate sulfotransferases. The carbohydrate sulfotransferases in red algae participate in the synthesis of sulfated polysaccharides.

In total, six genes are related to sulfurylases in *Gp. lemaneiformis* (Additional file 2: Table S2). Of these genes, four contigs (Contig1123.1, 137.1, 142.3 and 9907.5) belonged to the galactose-2, 6-sulfurylase I family, and two (Contig304.1 and 304.2) belonged to the galactose-2, 6-sulfurylase II family. Galactose-2, 6-sulfurylase specifically transfers aryl or alkyl groups to produce 3, 6-anhydrogalactose residues, which may play a key role in the conversion of the agar precursor to a mature polysaccharide in red algae. In *Chondrus*, 11 related sulfurylases have been identified, and eight were classified as type-II sulfurylases [23].

Sulfatases are necessary for sulfation modification in the cell walls of algae. Unexpectedly, no sulfatases were found in *C. crispus*, while nine genes encoding family 1 sulfatases were present in the brown alga *E. siliculosus* [24]. In the *Gp. lemaneiformis* genome, we found five sulfatase-encoding genes (Table S2). Of them, one (Contig5126.2) was an alkyl SULF, three contigs (Contig14346.1, 14,346.2 and 14,346.6) included arylsulfatases, and one (Contig14346.3) encoded N-acetylgalactosamine-6-sulfate sulfatase. Therefore, there are different modifications between agar and carrageenans, while some modifications are similar between agar and alginate.

#### Glycoside hydrolases (GHs)- and glycosyltransferases (GTs)-related enzymes

In total, 51 GH and 105 GT genes were identified in *Gp. lemaneiformis* (Additional files 3 and 4: Tables S3 and S4), which were fewer than those identified in the brown alga *Saccharina japonica* (130, 82) [25] but more than those identified in another red alga, *C. crispus* (65, 31) [23]. Seven GH families (i.e., GH19, GH25, GH28, GH42, GH43, GH53 and GH113) and three GT families (i.e., GT11, GT32 and GT61) were found only in *Gp. lemaneiformis*. Of these families, the GT61 family (11) consisted of 11 genes, while the other nine families each had only one copy. In contrast, several GH and GT families had different members in brown algae and red algae. For example, five GH families (i.e., GH10, GH17, GH30, GH88 and GH114) occurred only in *S. japonica*, and even more surprisingly, the GH81 family included 53 genes in *S. japonica* but only 1 and 0 in *Gp. lemaneiformis* and *C. crispus*, respectively. In addition, seven GT families (i.e., GT10, GT23, GT48, GT50, GT60, GT68 and GT74) were only present in *S. japonica*, and family GT23 consisted of 17 genes.

Trehalose, a kind of non-reducing disaccharide, is present in a wide variety of organisms including algae. The metabolic enzymes involved in trehalose include trehalase (family GH37), trehalose-phosphate synthase (TPS, family GT20), trehalose phosphatase and trehalose synthase. The first three trehalose-related enzymes were found in the *Gp. lemaneiformis* genome (Additional file 5: Table S5). In addition to one trehalose phosphatase gene, three trehalase and four TPS genes were detected in this alga, which are similar to those in *C. crispus* [23].

Cellulose is an important component of the cell walls of plants and algae. In total, 12 GT2 genes were identified in *Gp. lemaneiformis*, which was more than the number identified in *C. crispus* (3) and only half the number identified in *S. japonica* (24) (Additional file 3: Table S3). Of these genes, five GT2 genes belonged to the cellulose synthase superfamily, which has been classified into nine cellulose synthase-like (CSL) families and one cellulose synthase (CESA) family [26]. In addition to the three CESAs identified in the *Chondrus* genome, another CESA in Rhodophyta has been reported in *Porphyra yezoensis* and *Griffithsia monilis* [27, 28]. We identified five CESA or CSL genes in *Gp. lemaneiformis*. Similar to the CESAs in *Chondrus*, four CESAs were found in *Gp. lemaneiformis* (Contig33.5 was excluded because the amino acid sequence it encoded was short), and they were divided into two categories (Additional file 6: Figure S1). One class included CESAs from certain species of red algae, such as *Chondrus*, *Griffithsia*, *Porphyra*, and *Pyropia*, as well as two contigs (Contigs60.7 and 60.8) from *Gp. lemaneiformis*. Two other genes (Contig33.6 and 1420.1) clustered with the CESAs from prokaryotic organisms, such as *Nostoc* and *Bacillus*, and eukaryotic organisms from *Chondrus* and *Nannochloropsis*. Therefore, the plant CSLA and CSLC genes have different origins [29].

### Phytohormone signaling analysis

#### Auxins

IAA and indole-3-acetamide were identified as the main auxins in 11 red algae from the Brazilian coast [30]. In this work, multiple auxin-related genes were found, including auxin transport, auxin efflux carrier and auxin response factor genes (Additional file 7: Table S6). Of these genes, the indole-3-glycerol-phosphate synthases (4) were the most abundant. Moreover, the auxin transport protein BIG (2), auxin efflux carriers (2), and phenylacetic acid degradation proteins (2) were detected in *Gp. lemaneiformis*.

#### Abscisic acid

ABA-related components were also very enriched in this alga. Four 3′(2′), 5′-bisphosphate nucleotidases, which are components of the ABA-activated signaling pathway, were detected (Additional file 8: Table S7). Moreover, ABA-insensitive 5-like proteins (a total of 6) were found in the *Gp. lemaneiformis* genome. In addition to the key enzymes in ABA synthesis, such as 9-cis-epoxycarotenoid dioxygenase, one farnesylcysteine lyase, which is a negative regulator of ABA signaling [31], was also identified in *Gp. lemaneiformis*.

#### Salicylic acid

SA is a phytohormone that is involved in biotic and abiotic stress. Two SA synthesis pathways have been proposed in plants, including a pathway from cinnamate via the activity of phenylalanine ammonia lyase (PAL) and another pathway from isochorismate via two reactions catalyzed by isochorismate synthase (ICS) and isochorismate pyruvate lyase (IPL) in bacteria. *Arabidopsis* contains two ICS genes but no IPL genes [32]. Similar to *Arabidopsis*, one ICS gene but no IPL genes were detected in *Gp. lemaneiformis*. Additionally, as in *C. crispus*, genes encoding PAL and salicylate synthase were absent [23]. Moreover, one salicylate 1-monooxygenase and one chorismate mutase were detected in *Gp. lemaneiformis* (Additional file 9: Table S8).

#### Jasmonates

JAs, which include various lipid-derived signaling compounds, also play key roles in the growth and development of plants, as well as their stress and defense responses. A series of enzymes, including lipoxygenase (LOX), allene oxide synthase (AOS), allene oxide cyclase (AOC), and 12-oxophytodienoate reductase 3 (OPR3), participate in JA biosynthesis [33]. In higher plants, many enzymes involved in JA synthesis (e.g., six LOXs and four AOCs in *A. thaliana*) have been characterized [34, 35]. However, only two LOX genes, not AOS or AOC genes, were observed in the seaweed *C. crispus* [23]. Similar to *C. crispus*, two genes encoding LOX were detected in *Gp. lemaneiformis* (Additional file 10: Table S9). A pairwise comparison showed that the two LOX genes in this alga shared only 17.66% identity, which was similar to the 20% identity between the two LOX genes in *C. crispus*. The two LOX genes in *Gp. lemaneiformis* were separately distributed among the 14 LOX genes from 10 species in the phylogenetic tree (Additional file 11: Figure S2). Thus, the JAs in red algae might be synthesized through pathways unlike those in higher plants.

## Discussion

Currently, next-generation sequencing techniques, such as the Illumina and 454 platforms, are the main methods used to generate draft genomes. Using the RNA-seq technique, thegenomes of several algae such as *S. japonica* [25], *Porphyridium purpureum* [36], *Nannochloropsis gaditana* [37] have been constructed and elucidated. In 2013, a 97 Mb genome was reported, and 3490 genes were identified in the female gametophyte of the wild *Gp. lemaneiformis* [17]. Here, we present a draft genome of the tetrasporophyte of cultivated *Gp. lemaneiformis* strain 981 by combining RNA-seq and paired-end-tag (PET) approaches. Based on the size of the published wild *Gp. lemaneiformis* genome [17], approximately 91.73% of this algal genome was assembled in this study. The considerable number of repetitive elements is a major structural feature of plant genomes. For example, 73% of the genome of the macroalga *Chondrus crispus* (Rhodophyta) consists of repeated sequences [23]. This study indicated approximately 40.94% (38.73 Mb) repetitive elements in the cultivated *Gp. lemaneiformis*, however, 54.64% (44.35 Mb) TEs were revealed in the wild *Gp. lemaneiformis* [17]. But the GC contents in the two *Gp. lemaneiformis* genomes were identical (~48%) [17].

Agar is one of the most important polysaccharides in the cell walls of red algae, including in the genera *Gracilariopsis*, *Gracilaria* and *Gelidium*. An understanding of the agar biosynthetic enzymes in *Gp. lemaneiformis* is very important for improving its agar content for the seaweed industry. Although a pathway for agar biosynthesis in red algae has been proposed, it has not been fully elucidated [20, 21]. Several genes encoding galactose-1-phosphate uridylyltransferase, UDP-glucose pyrophosphorylase and phosphoglucomutase have been shown to be related to agar yield in *Gracilaria* and *Gracilariopsis* species [38–40]. As compared to green algae and brown algae, in red algae, the enzymes participating in agar biosynthesis are extremely abundant, and the agar synthesis-related genes in *Gp. lemaneiformis* were more enriched than those in the carrageenan red alga, *Chondrus*. Up to now, the metabolism of agar remains unclear. For example, the bypass from glucose-1-phosphate to GDP-D-mannose is unconvincing [20, 21]. In addition, Chang et al. [20] reported that UDP galactosyltransferase (**5)** and GDP galactosyltransferase (**10**) catalyzed the synthesis of the agar precursor, however, Lee et al. [21] recorded both of them as glycosyltransferase. In contrast to that in higher plants such as *Arabidopsis* and rice, the molecular information on red algal galactosyltransferases is limited [21].

In addition to agar biosynthetic enzymes, red algae can store other unique carbohydrates, such as floridean starch and floridoside, in addition to agar and carrageenan. Additionally, cellulose, mannan, xylans, and certain unique sulfated polysaccharides constitute a large fraction of the cell wall matrix [41]. The enzymes catalyzing the synthesis and breakage of glycosidic bonds in complex sugars, multiple GH and GT families, are discussed in this paper. The differences of the GH and GT families between the aforementioned species indicated that more GH and GT families are present in the brown algae *S. japonica* than those in the two red algae *C. crispus* and *Gp. lemaneiformis*; and more GH and GT families exist in *Gp. lemaneiformis* than in *C. crispus*.

Another disaccharide trehalose may serve as a signaling molecule in plants [42]. One product of TPS, i.e., trehalose-6-phosphate, is also recognized as a regulator of sugar metabolism in plants [43] and acts as an inhibitor of SnRK1 (SNF1-related protein kinase) activity. SnRK1 has been shown to be involved in the sucrose, starch and raffinose family oligosaccharide pathways [44, 45]. There are three different pathways and total four enzymes involved in the biosynthesis and degradation of trehalose. Except for trehalose synthase, which has been reported only in a few bacteria [46], other three trehalose enzymes were all found in the *Gp. lemaneiformis* genome.

Phytohormones are a large category of widely distributed chemicals in plants that not only regulate plant growth at low concentrations but also can act as signaling molecules that participate in defense reactions. Phytohormones exist not only in higher plants but also in marine algae [47, 48]. Although the physiological roles of phytohormones in algae are similar to those in higher plants, phytohormone biosynthetic pathways and metabolic mechanisms in algae remain elusive. In our work, we determined the contents of five phytohormones, IAA, ABA, JA, SA and cinnamic acid, in *Gp. lemaneiformis* using Gas Chromatography-Mass Spectrometer (GC-MS) [13]. Moreover, we found that exogenous 24-epibrassinolide could promote algal growth and agar synthesis in *Gp. lemaneiformis* under normal and high-temperature conditions [40]. Transcriptomic analyses showed that polar auxin transport, auxin signal transduction, and their crosstalk with other endogenous plant hormones, including zeatin, ABA, ethylene, SA, and brassinosteroids, were important in adventitious branch formation in *G. lichenoides* [49]. In this paper, we analyzed the components involved in phytohormone signaling pathways and identified numerous phytohormone-related genes.

## Conclusions

In the present study, we performed an 88.98 Mb draft genome assembly (approximately 91% of the entire genome size) and identified 9281 genes in the tetrasporophyte of the macroalga *Gp. lemaneiformis* (Rhodophyta). We then focused on carbohydrate metabolism, analyzing unique agar-synthesis enzymes and multiple GH, GT enzymes different from those of the *C. crispus* and *S. japonica* genomes. Finally, we summarized phytohormone signaling pathways, including those of auxin, ABA, SA and JAs, of which there is little research to date on red algae. The results will enhance our understanding of marine algal genomic information and might be helpful in the breeding and culture of this alga.

## Methods

### Sample preparation and DNA extraction

The tetrasporophytes of *Gp. lemaneiformis* strain 981 was collected at Ningde (26°65’N, 119°66′E), Fujian, China, on September 20, 2009. *Gp. lemaneiformis* 981 is cultured in large areas of the southeastern coast of China, and we received permission to collect the samples from the farmer. We removed epiphytes and maintained a unialgal culture at the algal collection center of Ningbo University. The algal thalli were subjected to DNA extraction using an HP Plant DNA Kit (Omega Bio-Tek, USA).

### Genome sequencing and assembly

The qualified genomic DNA was fragmented using the Covaris protocol, and fragments of 500 bp were used for paired-end library preparation using a KAPA Hyper Prep Kit (Kapa/Roche) according to the manufacturer’s instructions. The resulting library was sequenced on an Illumina HiSeq 2000 sequencer. The paired-end reads were used to assemble the contigs and then generate scaffolds using SOAPdenovo2 [50]. Sequences in the assembled contigs potentially from contaminating bacteria were removed by BLASTx searches against the NCBI non-redundant protein database.

### Transcriptome sequencing and analysis

Total RNA was extracted using the RNeasy Plant Mini Kit (Qiagen, Germany) following the manufacturer’s instructions. The RNA Library Prep Kit (NEB, USA) was used to generate the RNA-seq library, and paired-end sequencing was performed on an Illumina HiSeq 2000 sequencer. SOAPdenovo-Trans v1.03 was used to assemble the sequencing reads de novo using the default parameters [51].

### Gene prediction and annotation

We used RepeatModeler (http://www.repeatmasker.org/ RepeatModeler.html) to detect the TEs in the *Gp. lemaneiformis* genome using the default parameters. Prior to gene prediction, the detected TEs were removed from the assembled genome.

Then, homolog-based, ab initio and transcriptome-based approaches were integrated to predict the protein-coding genes in the *Gp. lemaneiformis* genome according to Ye et al. [25]. Furthermore, Augustus version2.5.5 [52] and GeneMarkES version 3.0.1 [53] were utilized for ab initio gene prediction. The program Exonerate v2.2.0 [54] was used with the protein sequences of *A. thaliana, C. merolae, P. tricornutum* and *C. crispus,* which were downloaded from Phytozome (http://www.phytozome.net), to perform homolog-based prediction. We mapped the RNA-seq data to the genome and assembled the transcripts to the gene models using TopHat and Cufflinks [55]. The gene models predicted from the abovementioned strategies were combined into a non-redundant consensus of gene structures using EvidenceModeler [56]. Finally, for the functional annotation of the protein-coding genes, BLASTp with a cut-off E-value of 1e-10 was performed against the NCBI non-redundant protein sequence database, and those genes with the best BLAST hits were combined into the gene set. The predicted genes were mapped to the KEGG protein database for metabolic pathway analysis.

## Additional files

Additional file 1: **Table S1.** Comparison of the number of enzymes involved in agar synthesis in the 6 species. (DOCX 26 kb)

Additional file 2: **Table S2.** The enzymes involved in converting the agar precursor into agar in *Gp. lemaneiformis*. (DOCX 24 kb)

Additional file 3: **Table S3.** The numbers of glycoside hydrolases (GHs) identified in the *Gp. lemaneiformis*, *C. crispus* and *S. japonica* genomes. (DOCX 26 kb)

Additional file 4: **Table S4.** The numbers of glycosyltransferases (GTs) identified in the *Gp. lemaneiformis*, *C. crispus* and *S. japonica* genomes. (DOCX 26 kb)

Additional file 5: **Table S5.** The enzymes involved in trehalose metabolism. (DOCX 24 kb)

Additional file 6: **Figure S1.** The phylogenetic relationship of cellulose synthase (CESA) in *Gp. lemaneiformis* with those of other related species. The tree was constructed based on the maximum likelihood method with 1000 bootstrap replicates using Mega5.10 software. (DOCX 27 kb)

Additional file 7: **Table S6.** The genes related to auxin signaling in *Gp. lemaneiformis*. (DOCX 24 kb)

Additional file 8: **Table S7.** The genes related to abscisic acid signaling in *Gp. lemaneiformis*. (DOCX 24 kb)

Additional file 9: **Table S8.** The enzymes related to salicylic acid signaling in *Gp. lemaneiformis*. (DOCX 25 kb)

Additional file 10: **Table S9.** The enzymes related to jasmonic acid signaling in *Gp. lemaneiformis*. (DOCX 24 kb)

Additional file 11: **Figure S2.** Phylogeny deduced from the lipoxygenases (LOX) of ten species. The tree was constructed based on the maximum likelihood method with 1000 bootstrap replicates using Mega5.10 software. (DOCX 24 kb)

## Abbreviations

ABA: Abscisic acid
AOC: Allene oxide cyclase
AOS: Allene oxide synthase
BP: Biological process
CC: Cellular component
CESA: Cellulose synthase
CSL: Cellulose synthase-like
GC-MS: Gas chromatography-mass spectrometer
GHs: Glycoside hydrolases
GO: Gene ontology
GTs: Glycosyltransferases
IAA: Indole-3-acetic acid
ICS: Isochorismate synthase
IPL: Isochorismate pyruvate lyase
JA: Jasmonic acid
JAs: Jasmonates
KEGG: Kyoto encyclopedia of genes and genomes
LINEs: Long interspersed repeated DNA elements
LOX: Lipoxygenase
LTR: Long terminal repeat
MF: Molecular function
MTs: Methyltransferases
OPR3: 12-oxophytodienoate reductase 3
PAL: Phenylalanine ammonia lyase
PET: Paired-end-tag
PTs: Pyruvyl transferases
RC/Helitron: Rolling-circle transposons
SA: Salicylic acid
SHs: Sulfohydrolases
SnRK: SNF1-related protein kinase
STs: Sulfotransferases
TEs: Transposable elements
TPS: Trehalose-phosphate synthase
