# Supplementary material for: Genomic analyses of unique carbohydrate and phytohormone metabolism in the macroalga Gracilariopsis lemaneiformis (Rhodophyta)
Source: BMC Plant Biol. 2018 May 25;18:94. doi: 10.1186/s12870-018-1309-2 (PMC5970526; doi:10.1186/s12870-018-1309-2)
Supplement: Supplementary file 1 — Table S1. Comparison of the number of enzymes involved in agar synthesis in the 6 species. (DOCX 26 kb) [file 12870_2018_1309_MOESM1_ESM.docx]

**Additional file 1**

**Table S1 Comparison of the number of enzymes involved in agar synthesis in the 6 species**

| **No.** | **Enzyme name** | ***Gp. lemaneiformis*** | ***C. merolae*** | ***C. reinhardtii*** | ***N.* gaditana** | ***E. siliculosus*** | ***P. tricornutum*** |
| --- | --- | --- | --- | --- | --- | --- | --- |
| 1 | phosphoglucose isomerase | 4 | 3 | 1 | 2 | 3 | 5 |
| 2 | phosphoglucomutase | 5 | 5 | 3 | 1 | 1 | 5 |
| 3 | UTP-glucose-1-phosphate uridylyltransferase | 2 | 1 | 4 | 0 | 0 | 1 |
| 4 | galactose-1-phosphate uridylyltransferase | 1 | 1 | 1 | 1 | 0 | 0 |
| 5 | UDP-galactosyltransferase | 0 | 0 | 0 | 0 | 0 | 0 |
| 6 | phosphomannose isomerase | 2 | 1 | 1 | 1 | 5 | 1 |
| 7 | phosphomannomutase | 6 | 3 | 2 | 0 | 3 | 2 |
| 8 | mannose-1-phosphate guanylyltransferase | 4 | 2 | 1 | 2 | 0 | 0 |
| 9 | GDP-mannose-3,5-epimerase | 1 | 1 | 1 | 0 | 0 | 0 |
| 10 | GDP galactosyltransferase | 0 | 0 | 0 | 0 | 0 | 0 |
| 11 | GDP-glucose pyrophosphorylase | 0 | 0 | 0 | 0 | 0 | 0 |
| 12 | GDP-mannose-3,5-epimerase | 1 | 1 | 1 | 0 | 0 | 0 |
