# Supplementary material for: Genomic analyses of unique carbohydrate and phytohormone metabolism in the macroalga Gracilariopsis lemaneiformis (Rhodophyta)
Source: BMC Plant Biol. 2018 May 25;18:94. doi: 10.1186/s12870-018-1309-2 (PMC5970526; doi:10.1186/s12870-018-1309-2)
Supplement: Supplementary file 2 — Table S2. The enzymes involved in converting the agar precursor into agar in Gp. lemaneiformis. (DOCX 24 kb) [file 12870_2018_1309_MOESM2_ESM.docx]

**Additional file 2**

**Table S2 The enzymes involved in converting the agar precursor into agar in *Gp. lemaneiformis***

| **Enzyme name** | **EC number** | **Gene ID** |
| --- | --- | --- |
| sulfotransferase | 2.8.2.- | Contig2022.7, 2652.1, 343.1 |
| glycolipid sulfotransferase | 2.8.2.- | Contig47.1, 6728.4 |
| carbohydrate sulfotransferase | 2.8.2.- | Contig145.10, 219.13, 4885.26 |
| galactose-2,6-sulfurylases I | 2.5.1.5 | Contig1123.1, 137.1, 142.3, 9907.5 |
| galactose-2,6-sulfurylases II | 2.5.1.5 | Contig304.1, 304.2 |
| alkyl sulfatase | 3.1.6.- | Contig5126.2 |
| arylsulfatase | 3.1.6.1 | Contig14346.1, 14346.2, 14346.6 |
| N-acetylgalactosamine 6-sulfatase | 3.1. 6.4 | Contig14346.3 |
| polysaccharide pyruvyl transferase | 2.-. -. - | Contig3012.14, 4766.16 |
| thiosulfohydrolase SoxB | 3.12.1.1 | Contig2543.10 |
