# Supplementary material for: Genomic analyses of unique carbohydrate and phytohormone metabolism in the macroalga Gracilariopsis lemaneiformis (Rhodophyta)
Source: BMC Plant Biol. 2018 May 25;18:94. doi: 10.1186/s12870-018-1309-2 (PMC5970526; doi:10.1186/s12870-018-1309-2)
Supplement: Supplementary file 3 — Table S3. The numbers of glycoside hydrolases (GHs) identified in the Gp. lemaneiformis, C. crispus and S. japonica genomes. (DOCX 26 kb) [file 12870_2018_1309_MOESM3_ESM.docx]

**Additional file 3**

**Table S3 The numbers of glycoside hydrolases (GH) identified in the *Gp. lemaneiformis*, *C. crispus* and *S. japonica* genomes**

|  | **GH1** | **GH2** | **GH3** | **GH5** | **GH6** | **GH10** | **GH13** | **GH14** | **GH16** |
| --- | --- | --- | --- | --- | --- | --- | --- | --- | --- |
| *Gp. lemaneiformis* | 1 | 2 | 1 | 2 | 2 | 0 | 6 | 1 | 7 |
| *C. crispus* | 2 | 0 | 0 | 1 | 2 | 0 | 4 | 1 | 3 |
| *S. japonica* | 1 | 3 | 1 | 2 | 0 | 1 | 1 | 0 | 3 |
|  | **GH17** | **GH18** | **GH19** | **GH25** | **GH28** | **GH30** | **GH31** | **GH35** | **GH36** |
| *Gp. lemaneiformis* | 0 | 3 | 1 | 1 | 1 | 0 | 4 | 3 | 0 |
| *C. crispus* | 0 | 0 | 0 | 0 | 0 | 0 | 2 | 2 | 2 |
| *S. japonica* | 2 | 1 | 0 | 0 | 0 | 1 | 0 | 0 | 0 |
|  | **GH37** | **GH38** | **GH42** | **GH43** | **GH45** | **GH47** | **GH63** | **GH77** | **GH81** |
| *Gp. lemaneiformis* | 3 | 1 | 1 | 1 | 0 | 6 | 1 | 1 | 1 |
| *C. crispus* | 1 | 1 | 0 | 0 | 3 | 4 | 1 | 1 | 0 |
| *S. japonica* | 1 | 0 | 0 | 0 | 0 | 2 | 0 | 0 | 53 |
|  | **GH85** | **GH88** | **GH97** | **GH113** | **GH114** | **GH128** |  |  |  |
| *Gp. lemaneiformis* | 1 | 0 | 1 | 1 | 0 | 1 |  |  |  |
| *C. crispus* | 1 | 0 | 0 | 0 | 0 | 0 |  |  |  |
| *S. japonica* | 1 | 1 | 0 | 0 | 7 | 1 |  |  |  |
