# Supplementary material for: Genomic analyses of unique carbohydrate and phytohormone metabolism in the macroalga Gracilariopsis lemaneiformis (Rhodophyta)
Source: BMC Plant Biol. 2018 May 25;18:94. doi: 10.1186/s12870-018-1309-2 (PMC5970526; doi:10.1186/s12870-018-1309-2)
Supplement: Supplementary file 4 — Table S4. The numbers of glycosyltransferases (GTs) identified in the Gp. lemaneiformis, C. crispus and S. japonica genomes. (DOCX 26 kb) [file 12870_2018_1309_MOESM4_ESM.docx]

**Additional file 4**

**Table S4 The numbers of glycosyltransferases (GT) identified in the *Gp. lemaneiformis*, *C. crispus* and *S. japonica* genomes**

|  | **GT1** | **GT2** | **GT4** | **GT5** | **GT7** | **GT8** | **GT10** | **GT11** | **GT13** |
| --- | --- | --- | --- | --- | --- | --- | --- | --- | --- |
| *Gp. lemaneiformis* | 3 | 12 | 14 | 1 | 5 | 0 | 0 | 1 | 1 |
| *C. crispus* | 0 | 3 | 3 | 1 | 4 | 2 | 0 | 0 | 2 |
| *S. japonica* | 3 | 24 | 13 | 0 | 1 | 5 | 2 | 0 | 1 |
|  | **GT14** | **GT20** | **GT22** | **GT23** | **GT24** | **GT25** | **GT27** | **GT28** | **GT31** |
| *Gp. lemaneiformis* | 1 | 4 | 1 | 0 | 2 | 5 | 2 | 4 | 2 |
| *C. crispus* | 9 | 4 | 1 | 0 | 1 | 0 | 2 | 4 | 0 |
| *S. japonica* | 1 | 4 | 2 | 17 | 0 | 1 | 1 | 4 | 4 |
|  | **GT32** | **GT33** | **GT34** | **GT35** | **GT39** | **GT41** | **GT45** | **GT47** | **GT48** |
| *Gp.lemaneiformis* | 1 | 0 | 3 | 1 | 5 | 2 | 0 | 2 | 0 |
| *C. crispus* | 0 | 1 | 1 | 1 | 6 | 1 | 1 | 3 | 0 |
| *S. japonica* | 0 | 0 | 1 | 0 | 0 | 4 | 0 | 14 | 2 |
|  | **GT49** | **GT50** | **GT57** | **GT58** | **GT59** | **GT60** | **GT61** | **GT64** | **GT66** |
| *Gp. lemaneiformis* | 2 | 0 | 2 | 1 | 0 | 0 | 11 | 2 | 1 |
| *C. crispus* | 0 | 0 | 2 | 1 | 1 | 0 | 0 | 2 | 1 |
| *S. japonica* | 1 | 1 | 2 | 1 | 0 | 1 | 0 | 3 | 0 |
|  | **GT68** | **GT74** | **GT77** | **GT78** | **GT90** | **GT92** |  |  |  |
| *Gp. lemaneiformis* | 0 | 0 | 7 | 1 | 4 | 2 |  |  |  |
| *C. crispus* | 0 | 0 | 5 | 1 | 2 | 0 |  |  |  |
| *S. japonica* | 2 | 1 | 11 | 0 | 1 | 2 |  |  |  |
