# Supplementary material for: Genomic analyses of unique carbohydrate and phytohormone metabolism in the macroalga Gracilariopsis lemaneiformis (Rhodophyta)
Source: BMC Plant Biol. 2018 May 25;18:94. doi: 10.1186/s12870-018-1309-2 (PMC5970526; doi:10.1186/s12870-018-1309-2)
Supplement: Supplementary file 5 — Table S5. The enzymes involved in trehalose metabolism. (DOCX 24 kb) [file 12870_2018_1309_MOESM5_ESM.docx]

**Additional file 5**

**Table S5 The enzymes involved in trehalose metabolism**

| **Enzyme name** | **EC number** | **Gene ID** |
| --- | --- | --- |
| trehalose-phosphate synthase (TPS) | 2.4.1.15 | Contig4915.8, 18.3, 174.1, 214.2 |
| trehalose phosphatase | 3.1.3.12 | Contig253.6 |
| trehalase | 3.2.1.28 | Contig793.1, 2904.64, 46789.1 |
| trehalose synthase | 2.4.1.245 | Not found |
