# Supplementary material for: Genomic analyses of unique carbohydrate and phytohormone metabolism in the macroalga Gracilariopsis lemaneiformis (Rhodophyta)
Source: BMC Plant Biol. 2018 May 25;18:94. doi: 10.1186/s12870-018-1309-2 (PMC5970526; doi:10.1186/s12870-018-1309-2)
Supplement: Supplementary file 7 — Table S6. The genes related to auxin signaling in Gp. lemaneiformis. (DOCX 24 kb) [file 12870_2018_1309_MOESM7_ESM.docx]

**Additional file 7**

**Table S6 The genes related to auxin signaling in *Gp. lemaneiformis***

| **Gene name** | **Gene ID** |
| --- | --- |
| auxin transport protein BIG | Contig392.1, 2913.27 |
| auxin efflux carrier | Contig368.4, 502.6 |
| auxin-responsive family protein | Contig2547.18 |
| auxin response factor 2 | Contig3176.1 |
| auxin response factor 3 | Contig29901.1 |
| auxin responsive GH3 family protein | Contig283.4 |
| auxin resistant 1 | Contig719.1 |
| auxin-induced protein 5NG4 | Contig5764.1 |
| indole-3-glycerol phosphate lyase | Contig1.11 |
| indole-3-glycerol-phosphate synthase | Contig1051.1, 220.2, 350.1, 490.6 |
| phenylacetic acid degradation protein | Contig 4378.5, 2733.1 |
| phenylacetic acid catabolic family protein | Contig484.4 |
