# Supplementary material for: Genomic analyses of unique carbohydrate and phytohormone metabolism in the macroalga Gracilariopsis lemaneiformis (Rhodophyta)
Source: BMC Plant Biol. 2018 May 25;18:94. doi: 10.1186/s12870-018-1309-2 (PMC5970526; doi:10.1186/s12870-018-1309-2)
Supplement: Supplementary file 8 — Table S7. The genes related to abscisic acid signaling in Gp. lemaneiformis. (DOCX 24 kb) [file 12870_2018_1309_MOESM8_ESM.docx]

**Additional file 8**

**Table S7 The genes related to abscisic acid signaling in *Gp. lemaneiformis***

| **Gene name** | **Gene ID** |
| --- | --- |
| SnRK2.3 | Contig2835.1 |
| 3'(2'), 5'-bisphosphate nucleotidase | Contig32954.134, 2817.3, 2129.2, 40.5 |
| farnesylcysteine lyase | Contig5484.4 |
| 9-cis-epoxycarotenoid dioxygenase | Contig10994.43 |
| abscisic acid- insensitive 5-like protein 2 | Contig2756.22, 5064.18, 4915.5 |
| abscisic acid-insensitive 5-like protein 5 | Contig4992.1 |
| abscisic acid-insensitive 5-like protein 6 | Contig9907.3 |
| abscisic acid-insensitive 5-like protein 7 | Contig84.32 |
| abscisic acid G-protein coupled receptor-like domain-containing protein | Contig2652.8 |
