# Supplementary material for: Genomic analyses of unique carbohydrate and phytohormone metabolism in the macroalga Gracilariopsis lemaneiformis (Rhodophyta)
Source: BMC Plant Biol. 2018 May 25;18:94. doi: 10.1186/s12870-018-1309-2 (PMC5970526; doi:10.1186/s12870-018-1309-2)
Supplement: Supplementary file 9 — Table S8. The enzymes related to salicylic acid signaling in Gp. lemaneiformis. (DOCX 25 kb) [file 12870_2018_1309_MOESM9_ESM.docx]

**Additional file 9**

**Table S8 The enzymes related to salicylic acid signaling in *Gp. lemaneiformis***

| **Enzyme name** | **EC number** | **Gene ID** |
| --- | --- | --- |
| salicylic acid carboxyl methyltransferase-like protein | 2.1.1.274 | Contig3053.1 |
| salicylate 1-monooxygenase | 1.14.13.1 | Contig67.6 |
| phenylalanine ammonia-lyase (PAL) | 4.3.1.24 | Not found |
| isochorismate synthase (ICS) | 5.4.4.2 | Contig4364.45 |
| anthranilate synthase | 4.1.3.27 | Contig5558.7, 33654.109, 3220.1 |
| chorismate mutase | 5.4.99.5 | Contig51.34 |
| salicylate synthase | 4.3.1.- | Not found |
| aminodeoxychorismate synthase | 2.6.1.85 | Contig4942.1, 5561.11, 14346.17 |
