# Supplementary material for: Genomic analyses of unique carbohydrate and phytohormone metabolism in the macroalga Gracilariopsis lemaneiformis (Rhodophyta)
Source: BMC Plant Biol. 2018 May 25;18:94. doi: 10.1186/s12870-018-1309-2 (PMC5970526; doi:10.1186/s12870-018-1309-2)
Supplement: Supplementary file 10 — Table S9. The enzymes related to jasmonic acid signaling in Gp. lemaneiformis. (DOCX 24 kb) [file 12870_2018_1309_MOESM10_ESM.docx]

**Additional file 10**

**Table S9 The enzymes related to jasmonic acid signaling in *Gp. lemaneiformis***

| **Gene name** | **EC number** | **Gene ID** |
| --- | --- | --- |
| lipoxygenase (LOX) | 1.13.11.12 | Contig2900.3, 2207.11 |
| allene oxide synthase (AOS) | 4.2.1.92 | Not found |
| allene oxide cyclase (AOC) | 5.3.99.6 | Not found |
| 12-oxophytodienoate reductase3 (OPR3) | 1.3.1.42 | Not found |
