# Supplementary material for: Genomic analyses of unique carbohydrate and phytohormone metabolism in the macroalga Gracilariopsis lemaneiformis (Rhodophyta)
Source: BMC Plant Biol. 2018 May 25;18:94. doi: 10.1186/s12870-018-1309-2 (PMC5970526; doi:10.1186/s12870-018-1309-2)
Supplement: Supplementary file 10 [file 12870_2018_1309_MOESM11_ESM.docx]

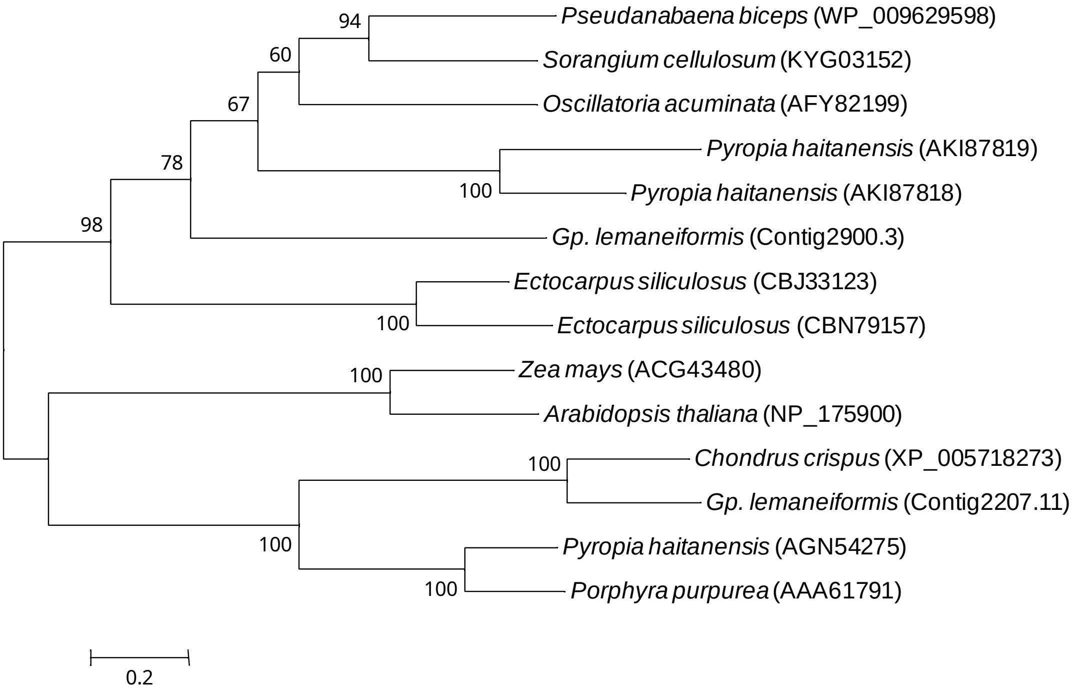

Pseudanabaena biceps (WP_009629598)
Sorangium cellulosum (KYG03152)
Oscillatoria acuminata (AFY82199)
Pyropia haitanensis (AKI87819)
Pyropia haitanensis (AKI87818)
Gp. lemaneiformis (Contig2900.3)
Ectocarpus siliculosus (CBJ33123)
Ectocarpus siliculosus (CBN79157)
Zea mays (ACG43480)
Arabidopsis thaliana (NP_175900)
Chondrus crispus (XP_005718273)
Gp. lemaneiformis (Contig2207.11)
Pyropia haitanensis (AGN54275)
Porphyra purpurea (AAA61791)
94
60
67
78
98
100
0.2
