# Supplementary material for: Genomic analyses of unique carbohydrate and phytohormone metabolism in the macroalga Gracilariopsis lemaneiformis (Rhodophyta)
Source: BMC Plant Biol. 2018 May 25;18:94. doi: 10.1186/s12870-018-1309-2 (PMC5970526; doi:10.1186/s12870-018-1309-2)
Supplement: Supplementary file 11 [file 12870_2018_1309_MOESM6_ESM.docx]

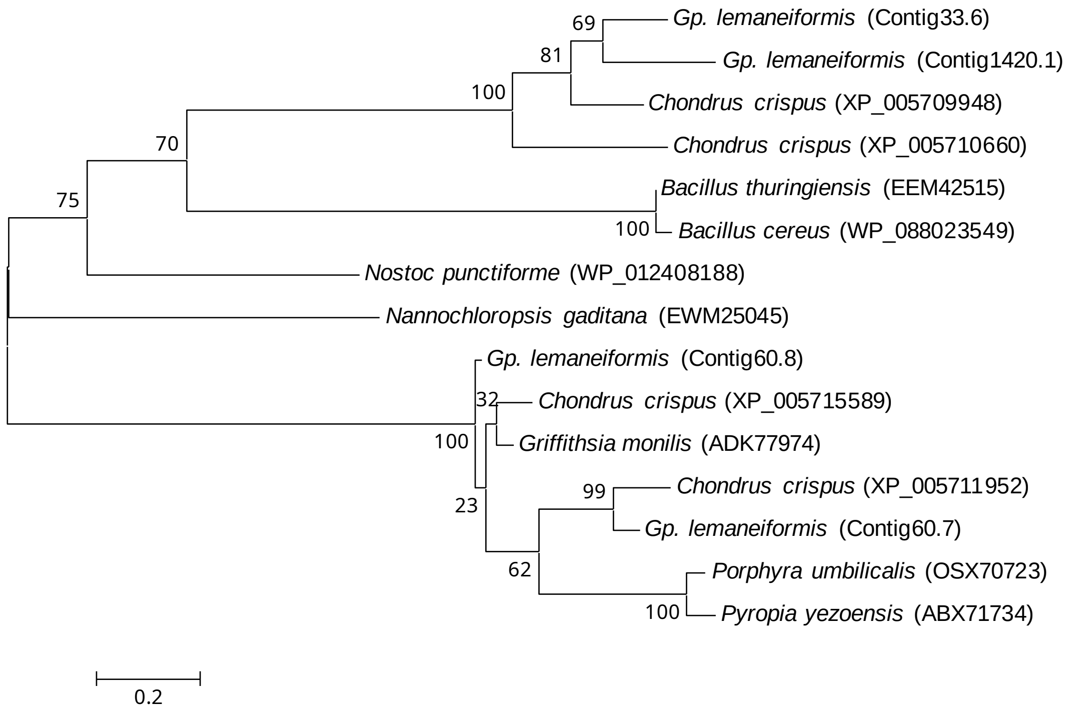
69
Gp. lemaneiformis (Contig33.6)
81
Gp. lemaneiformis (Contig1420.1)
100
Chondrus crispus (XP_005709948)
70
Chondrus crispus (XP_005710660)
75
Bacillus thuringiensis (EEM42515)
100
Bacillus cereus (WP_088023549)
Nostoc punctiforme (WP_012408188)
Nannochloropsis gaditana (EWM25045)
Gp. lemaneiformis (Contig60.8)
32
Chondrus crispus (XP_005715589)
100
Griffithsia monilis (ADK77974)
99
Chondrus crispus (XP_005711952)
23
Gp. lemaneiformis (Contig60.7)
62
Porphyra umbilicalis (OSX70723)
100
Pyropia yezoensis (ABX71734)
0.2
